# Oncotype DX Breast Cancer recurrence score resists inter-assay reproducibility with RT^2^-Profiler Multiplex RT-PCR

**DOI:** 10.1038/s41598-019-56910-0

**Published:** 2019-12-30

**Authors:** Verena Schildgen, Mathias Warm, Michael Brockmann, Oliver Schildgen

**Affiliations:** 1 Kliniken der Stadt Köln gGmbH, Klinikum der Privaten Universität Witten/Herdecke, Institut für Pathologie, Ostmerheimer Str. 200, D-51109 Köln, Germany; 2 Kliniken der Stadt Köln gGmbH, Brustzentrum, Neufelder Str. 34, D-51067 Köln, Germany

**Keywords:** Tumour biomarkers, Breast cancer

## Abstract

The Oncotype Dx assay is frequently used to test if breast cancer patients can be spared from chemotherapy without negative effects for their future clinical course. However, due to conflicting data on the assay utility, in the recent past its reimbursement situation in Germany was revised; due to continued requests by clinicians for predictive values, our group decided to implement an Oncotype Dx like alternative assay with the objective of obtaining quality and cost optimization. Customized RT^2^-Profiler assays covering the 21 gene panel of the Oncotype Dx assay were applied to a pilot cohort of breast cancer patients with known Oncotype Dx Recurrence Score (RS). The Ct values obtained with RT^2^-Profiler-assays were used to calculate the unscaled Recurrence Score (RSu) values and the thereon based RS according to the Oncotype DX assay rules if available. Despite consistent assay performance it was impossible to establish correlations between RT^2^-Profiler recurrence scores with the respective Oncotype DX values not to mention exact matches. By following the Oncotype DX assay and its interpretation as close as possible we faced several obstructions such as lack of information on RNA amount used, missing units in the single gene expression report, missing references cited in the original study that should explain the determination of the recurrence score formula, and vague information on the normalization of the gene expression impeding the reproduction of Oncotype Dx results in other laboratories. Unfortunately, the Oncotype Dx assay cannot be confirmed by the customized RT^2^-profiler assay, not least because of the fact that the individual gene measurements are not provided in the medical report, although these are mandatory for the RS calculation. In fact, the “single gene report” only contains unscaled scores of the ER, PR, and Her2 genes without any internationally accepted unit used to describe a transcript quantity. Therefore a direct comparison with the in-house measurement to evaluate its performance is impossible. With regard to our findings and the fact that the Oncotype RS represents a likelihood of the risk of relapse it thus remains impossible to assess the clinical necessity of this assay.

## Introduction

In 2004 a study published by Paik and coworkers reported the utility of a new prognostic gene expression assay in breast cancer patients^1^. Since then the Oncotype Dx assay, which is aimed to identify the risk of recurrence if chemotherapy is not applied for node negative breast cancer patients, is broadly requested by senology professionals.

In the year 2014 Timothy Errington and his coworkers published an open investigation in which they analyzed the reproducibility of cancer biology research^2^. The authors addressed the question if key experiments from 50 cancer studies published between 2010 and 2012 in so called high impact journals could be replicated. Therefore they expected that the two key features of science, reproducibility and transparency, would apply to those studies as these generally accepted key features can be seen as a prerequisite for modern science^2^.

In 2017 two authors of the *Cancer Biology Reproducibility Study*, Nosek and Errington, published the first preliminary results^3^. Whilst they concede that “there is no such thing such as exact replication because there are always differences between the original study and the replication”, they claimed that retesting a hypothesis with the same or a closely related methodology should provide the same evidence enabling the investigators to converge on a general explanation independent of methodology^3^.

Forced by continued requests for the Oncotype DX Recurrence Score despite discontinued reimbursement by health insurances, we decided to develop an in-house assay that could be used to reproduce the Oncotype DX breast cancer scoring methodology in our hospital, although it is stated that technical reproduction is only possible in the core lab facility. Thereby we wanted to test the hypothesis that a high RS reported by Genomic Health would correspond to a high RS obtained with RT^2^-Profiler Ct-values if applied to the Oncotype DX RS calculation formula, whilst a low Oncotype Dx RS should be confirmed by the RT^2^-Profiler assay data as well.

## Materials and Methods

### Study design, a priori power analysis, and samples

Before the pilot study was initiated, an a priori case number calculation was performed. It was hypothesized that by using an in house RT^2^-Profiler assay the Oncotype DX RS could be replicated, most likely by including an adjustment based on a linear regression, implying that a high Oncotype score was expected to correspond to a high RT^2^-Profiler score, whilst a low Oncotype score should correspond to a low RT^2^-Profiler score.

The a priori power analysis was performed with the statistic’s software nQuery 4.0 and the following settings. Based on the assumption, that the Oncotype Dx method is fully published it was hypothesized that a similar qPCR method must deliver highly similar results as typical for diagnostic assays. Therefore, it was assumed that a reproducibility rate of 95% should be reached in the direct comparison of both methods. Thereby, a linear correlation between both assays also would be acceptable, as both methods are highly similar, but not equal. Taking into account an RS reproducibility of the Oncotype Dx assay of +/−2.2 counts, the maximum deviation between two measurements would be 4.4 RS counts, rounded 5 counts. In order to confirm a difference between both assays of 5 RS counts with an assumed standard deviation of 10 RS counts in a two-sided 99% confidence interval, a minimum of 27 cases has to be investigated.

For this reason we included 32 patients suffering from invasive carcinoma of non-specific type (NST) into a pilot study, who were previously tested for the Oncotype Dx RS between January 2016 and December 2017. The basic data from the Oncotype Dx assay, immunohistochemistry of the patient cohort, RS values predicted by the Breast Cancer Recurrence Score Estimator (http://www.breastrecurrenceestimator.onc.jhmi.edu/), and variation coefficients of RT-Profiler gene expression are summarized in Tables 1 and 2.

**Table 1:** Overview of the variation coefficients regarding the 21 gene transcripts within a single qPCR plate and between two independent plates.

Genes	Variation coefficient	
	Intra plate	Inter plate
ESR1 (ER)	3.12%	3.30%
BCL2	1.55%	1.13%
BIRC5 (Survivin)	3.34%	1.77%
ERBB2 (Her2)	2.36%	5.63%
CTSL2	<0.01%	<0.01%
GSTM1	5.02%	5.14%
GUSB	1.01%	1.01%
RTC (internal assay control)	0.98%	1.47%
PGR	2.86%	6.82%
MKI67 (Ki67)	4.51%	4.35%
CCNB1	5.24%	4.28%
GRB7	2.32%	3.41%
CD68	7.88%	3.40%
GAPDH	1.35%	1.06%
TFRC	2.68%	3.24%
PPC (internal assay control)	0.69%	0.63%
SCUBE2	1.97%	1.87%
AURKA (STK15)	3.21%	4.69%
MYBL2	3.99%	1.71%
MMP11	1.00%	1.45%
BAG1	1.49%	0.85%
RPLP0	0.82%	2.96%
ACTB	4.08%	4.78%
GDC(internal assay control)	<0.01%	<0.01%
mean	2.67%	2.83%
median	1.62%	1.74%

**Table 2:** Patient characteristics. Oncotype Dx scores including ER, PR, and Her2 scores as well as immunohistochemistry were available for all patients. In addition, the unrestricted RT^2^-Profiler Ct values ≤ 35 for ER, PGR, and Her2 are shown, Ct values > 35 are defined as negative.

patient	Oncotype Dx Score	ER Score (GHI)	ER IHC [% pos. cells]	unrestricted RT^2^- Ct-value	PR Score (GHI)	PGR IHC [% pos. cells]	unrestricted RT^2^-Ct-value	Her2 Score (GHI)	Her2 IHC [% pos. cells]	unrestricted RT2-Ct-value	Ki67 IHC [% pos. cells]	unrestricted RT2-Ct-value	predicted score*
1	29	12.4	100	31.6	<3.2	2	—	9.1	1	—	19	31	UD
2	10	11.7	80	—	8	30	—	9.9	1	—	33	—	UD
3^#^	30	10.9	100	30.6	4.2	100	34.6	9.4	1	32.7	10	31.5	LR
5	14	9.7	100	33.1	7.8	100	29.1	9.8	30	—	31	30	LR
6^#^	8	10.7	90	24.4	8.4	0	25.3	9.7	30	26.5	36	25.9	UD
7	18	9.2	98	—	7.8	95	33.3	9	1	—	14	—	LR
8	29	8	100	—	5.3	100	—	9.7	40	—	13	—	LR
10	12	10.8	90	—	7.8	70	—	9.2	1	—	23	—	LR
11	23	10.6	100	30	7.3	100	31.3	9.8	1	31.8	10	29.9	LR
12	6	10.5	90	28.6	8.9	80	27.6	8.7	1	30.7	21	28.5	LR
14	15	9.4	100	26.6	9.1	100	24.5	9	15	27.7	25	26.8	LR
15	39	8.9	100	—	3.7	100	—	9.8	1	—	22	—	LR
16	15	9.2	60	31.8	8.5	80	29.5	8.7	30	32.2	21	29.9	LR
17	36	11.2	100	27.7	6	100	—	9.8	30	29.5	12	27.9	LR
19	21	11.3	95	30.9	8.5	70	29.6	8.7	30	32.2	36	29.7	LR
20	23	8.2	100	28.6	6.7	100	28.1	9.4	40	28.6	13	29.5	LR
21	17	11.2	100	—	8.4	95	—	8.3	1	—	25	—	LR
22	13	10.2	100	31	8.6	100	28.9	9.2	40	32.8	20	30.2	LR
23	35	11.1	90	29.8	4.1	1	34.8	10.7	40	31.2	31	29.5	UD
24	13	10.1	80	—	7.4	80	—	9.6	1	—	10	—	UD
25	34	10.1	95	27.8	6.7	40	29.3	8.1	40	33.2	45	—	LR
26	34	11.4	100	26	7.7	100	26.7	9	1	—	30	27	LR
27	19	9.9	100	—	7.6	70	32.9	8.7	40	—	20	—	LR
28	14	10.2	90	27	7.7	80	26.7	8.4	1	31	26	28.4	LR
29	15	10.2	70	31	8.6	40	30.9	10.4	40	31.1	20	31.1	LR
30	20	10.4	100	27.9	8.6	95	26.7	9.3	30	29.9	27	28.1	LR
31^#^	28	7.9	90	—	5.5	40	—	9.4	30	—	17	—	LR
32^#^	4	11.8	100	30.6	9.1	85	30.9	10.1	10	33.2	42	32.1	LR
I^#^	26	12.1	60	31.3	4.4	75	—	9.7	10	33.7	20	31.2	LR
II^#^	11	10.2	90	33.9	6.8	45	—	8.7	10	—	4	33	LR
III	20	9.2	90	32.5	6.6	90	32.1	9.3	10	34.3	7	32.9	LR
IV	19	10.1	90	27.9	7.3	60	29.5	8.6	30	30.2	24	27.9	LR

IHC results of Ki67 were used to determine the recurrence risk with the Breast Cancer Recurrence Score (BCRS) Estimator (http://www.breastrecurrence estimator.onc.jhmi.edu). The comparison of the Oncotype Dx single gene scores with the RT^2^-Profiler Ct values as well as the comparison of the Oncotype Dx RS with the BCRS did not reveal any correlation. UD = undetermined, LR = low risk.

^#^Patient I = A, Patient II = B, Patient 3 = C, Patient 6 = D, Patient 32 = E, Patient 31 = F.

*The prediction was performed using the Breast Cancer Recurrence Score Estimator (http://www.breastrecurrenceestimator.onc.jhmi.edu).

The medical report from Genomic Health as well as the original FFPE tissue previously sent to and returned from Genomic Health was available for all patients. Within this patient cohort the age ranged from 39 years up to 75 years divided into subgroups of <40 year old (3,1%), 40 to 60 year old (68,8%), and >60 year old (28,1%). Due to data protection considerations we are not allowed to publish patient specific clinical data sets.

Of those specimens, two randomly selected FFPE tumor blocks were double pseudonymized and sent to Genomic Health for retesting.

### Ethical statement

All procedures were carried out in accordance with relevant national and international guidelines such as the declaration of Helsinki in its present form. The study was approved by the local ethical committee from the University of Witten (vote number 65/2018 from 22.05.2018). The ethical committee agreed that due to the retrospective character of the study and the pseudonymization no written informed consent from the patients was required.

### RNA extraction

RNA was extracted from macrodissected FFPE tumor tissues with the RNeasy FFPE kit from Qiagen strictly following the protocol of the manufacturer (Qiagen, Hilden, Germany) including a second DNase digestion step if necessary. Macrodissection was performed on advice of a pathologist, who also determined the tumor content in advance to RNA extraction.

### RT^2^-Profiler Assays

There are two ordering types of RT^2^-profiler assays: the predesigned assays focusing on distinct pathways or specific entities and the customized assays, for which genes can be freely selected from the overall gene offer. The latter assay type simply allows a customized combination of primer sets, which are already integrated in the predesigned assays. Thus, the entire RT^2^-profiler procedure used in the present study is commercially available and all qPCRs are pretested with warranted high sensitivities and specificities if the entire RT^2^-Profiler technology is used. Thus, even though the exact primer sequences remain confidential by the manufacturer, the customized RT^2^-profiler assay used in this study can be ordered with the identical quality from any lab worldwide by making use of the customized array service (https://www.qiagen.com/de/geneglobe) (supplement 1).

**Figure 1:**
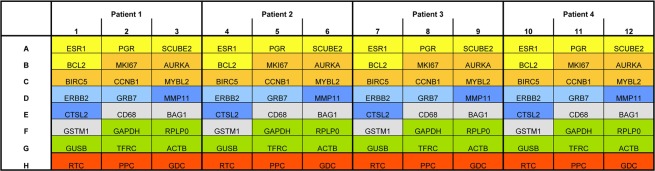
Overview on the plate layout of the RT^2^-Profiler assays.

Regarding the target genes the RT^2^-Profiler assays were designed in analogy to the Oncotype DX breast cancer assay. All targets were standard targets of the RT^2^-Profiler portfolio, which were just newly combined to emulate the Oncotype DX breast cancer assay. Controls detecting artificial DNA sequences (PCR Positive Control, PPC), cDNA reverse transcribed from RNA (Reverse Transcription Control, RTC), as well as a DNA contamination control (Genomic DNA Control, GDC) were included in the assay for each sample as recommended by the manufacturer (Qiagen, Hilden, Germany). The RT^2^-Profiler plate’s layout was designed to test 4 samples simultaneously as shown in Fig. 1 and supplement 1. Before testing the complete patient cohort, intra- and inter-assay reproducibility was determined by analyzing RNA samples (100 ng each) of two randomly selected patients (I = A and II = B) in quadruplicate on the same and on different RT^2^-Profiler plates, as well as two additional independent patient samples (III and IV). The assays were strictly performed according to the manufacturer’s instructions (Qiagen, Hilden, Germany) and only those assays that fulfill the specification GDC ≥ 35 and PPC 20 ± 2 cycles were included in the study.

In brief, cDNA synthesis was performed by incubating the genomic DNA elimination mix containing 0.1/0.5 µg RNA for 5′ at 42 °C followed by the reverse transcription reaction for 30′ at 42 °C, which is terminated by incubation for 5′ at 95°. Cycling conditions of the pre-amplification recommended for FFPE samples were: 1 cycle of 10′ at 95 °C and 8 cycles of 15” at 95 °C and 2′ at 60 °C, according to the RT^2^ PreAmp cDNA synthesis protocol (Qiagen, Hilden, Germany). Before starting the RT^2^-Profiler array samples were incubated for 15′ at 37 °C followed by 5′ at 95 °C. The analysis itself was performed on a LC480 instrument (Roche, Mannheim, Germany), with the following PCR conditions: 1 cycle for 10′ at 95 °C (ramp rate 4.4 °C/s), followed by 45 cycles of 15” at 95 °C and 1′ at 60 °C (ramp rate 1.5 °C/s). The detailed layout and ordering information of the individual qPCRs included in the custom RT^2^-Profiler assay are summarized in the supplementary file 1, which was kindly provided by Dr. Adile Acarkan, Senior Sales Application Specialist (Central Europe (Austria, Germany, Switzerland), Qiagen, Hilden, Germany).

### Calculation of the group scores and the Recurrence Score

The different RT^2^-Profiler group scores and the respective RS were determined as described by Paik *et al*.^1^ and the patent’s documents (International Patent Publication Numbers WO2006/052862A1 and WO2014/130617A1). The Ct values used for the calculation were obtained from the RT^2^-Profiler assays.

## Results

### Setup of Assay quality standards

In order to recreate an Oncotype-like in-house assay for non-commercial usage in our routine diagnostic laboratory a customized RT^2^-Profiler assay in 96-well format was designed (Fig. 1). The assay targets the same breast-cancer related and housekeeping genes as the Oncotype Dx assay and in addition a reverse transcription control (RTC), a genomic DNA control (GDC), and a positive PCR control (PPC) were performed.

**Figure 2:**
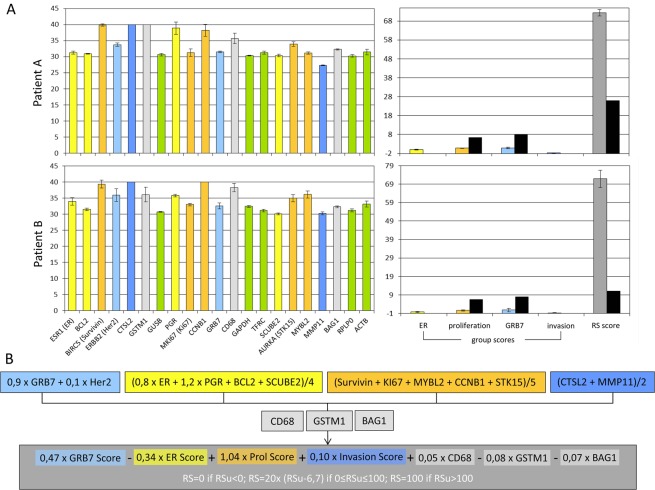
Intra- and inter-assay reproducibility of customized RT^2^-Profiler assays for the quantitative analyses of 21 gene transcripts according to the Oncotype Dx assay. The patients A and B were randomly selected. After RNA extraction, RT^2^-Profiler assays were repeatedly performed with 100 ng RNA on the same plate (intra-assay reproducibility) and on different plates (inter-assay reproducibility) according to the manufacturer’s protocol (Customized RT^2^-Profiler array, Qiagen, Hilden, Germany). For both patients the individual gene expression profiles as unrestricted Ct-values and the group scores are shown (2a). Thereby, the colours of the single genes correspond to the respective scores; housekeeping genes are coloured in green. The scores (ER = yellow, PR = orange, Her2 = light blue, Invasion = blue) were calculated exactly according to the formula (2b) published previously^1^. Black bars indicate the thresholds predefined by Genomic Health.

Initially, the inter-assay and intra-assay variability and reproducibility were determined. For this reason, two randomly selected patient samples (Patient A and Patient B) were repeatedly tested on the same RT^2^-Profiler plate as well as on different RT^2^-Profiler plates in independent, sequentially performed experiments (Fig. 2a). Thereby the standard deviations of the mean were calculated for all individual genes, the group scores and the RS (Fig. 2a), the latter calculated as previously described by Paik *et al*.^1^ (Fig. 2b). Housekeeping genes (HKGs) are colored in green, genes used to calculate the GRB7 group score are colored in light blue, genes belonging to the invasion group score are colored in blue, genes used for the ER group score are colored in yellow, and genes used to calculate the proliferation group score are colored in orange (Fig. 2).

The maximum standard deviation (SD) observed from mean Ct value was 2.28 for GSTM1 in one evaluation sample, whereas the averaged SD of the controls were 0.09 for PPC and 0.16 for RTC and of all analyzed genes 0.73 (Patient A) and 0.58 (Patient B), Thus, the in house assay had a good intra- and inter-assay reproducibility. The overall variation coefficients of within-laboratory repeatability ranged from <0.01% to 7.88% and were highest for CD68 and lowest for CTSL2. The differences between two runs of the identical specimen on a single plate displayed variation coefficients between <0.01% (CTSL2) and 7.88% (CD68) (mean 2.67%, median 1.62%), whilst between two independent plates the coefficients of variation ranged between <0.01% (CTSL2) and 6.82% (PGR) (mean 2.83%, median 1.74%). The individual coefficients are summarized in Table 1.

For these 8 RT^2^-profiler evaluation analyses as well as for two additional patient samples (III and IV) 100 ng of RNA were used. This RNA amount is within the range recommended by the manufacturer and should represent the minimal quantity requirements of diagnostic FFPE specimen. As we did not observe any correlation between the Oncotype RS scores and the scores calculated with the Ct values gained from these 10 data sets, the amount of RNA used was increased to 500 ng, according to the publication by Paik and coworkers, who identified samples containing less than 500 ng RNA as inappropriate. However, it cannot be excluded that the RNA amount used for the Oncotype DX has to be significantly higher than 500 ng as no information regarding this issue is provided.

### Patient expression data

The average Ct value for the five reference genes was 29.1 considering the data of all patients and 28.0 if one leaves out patients with less than five reference genes detected. Regarding the ER (estrogen receptor) expression all samples were characterized as ER positive by the Oncotype Dx ER score, whereas ER RNA was detected by the RT^2^-Profiler assay in 71.9% although all patients tested ER positive by IHC (Tables 2 and 3). This discrepancy is not surprising because half-life of RNA and proteins may deviate, especially in FFPE specimen, in so far as the protein is still detectable, although the RNA is degraded. This phenomenon is also reflected by the Oncotype Dx ER scores which range in samples with 100% IHC positivity from 8 (sample 8) to 12.4 (sample 1), whereas the Oncotype Dx ER score of sample I is 12.1 at an IHC positivity of only 60% (Table 2). In case of Her2 the respective Oncotype Dx single scores remained below the threshold of 10.7 except one equivocal sample * (Table 3). With the RT^2^-Profiler assay Her2 RNA was detected in 59.4% of the analyzed samples, whereas the other samples remained below the threshold. The RT^2^-Profiler results correspond to 71.9% to IHC, whereas the Oncotype Dx Her2 scores match with 37.5% of the IHC results. In five cases the RT^2^-Profiler as well as the Oncotype Dx scores were negative despite a positive IHC (Table 3). Of the 32 samples 21 (65.6%) tested PGR (progesterone receptor) positive and 11 (34.4%) tested PGR negative by the RT^2^-Profiler assay. Compared to the Oncotype Dx PR scores there is an inter-assay accordance regarding PGR-IHC of about 72% including the finding that both tests identified sample 6 as positive although it was IHC negative and that both tests identified three samples with 100% and 75% PGR-IHC positivity, respectively, as negative. BIRC expression was detected only in two samples and in 6 samples none of the Proliferation group score genes were detected at all. IHC results of Ki67 were used to determine the recurrence risk with the Breast Cancer Recurrence Score (BCRS) Estimator. Table 2 shows that the comparison of the Oncotype Dx single gene scores with the RT^2^-Profiler Ct values as well as the comparison of the Oncotype Dx RS with the BCRS did not reveal any reliable correlation.

**Table 3:** Comparison of IHC results for ER, PGR, and Her2 with respective RT^2^-Profiler RNA expression values and Oncotype DX singe gene scores.

patient	ER Score (GHI)	ER IHC [% pos. cells]	unrestricted Ct-value	PR Score (GHI)	PGR IHC [% pos. cells]	unrestricted Ct-value	Her2 Score (GHI)	Her2 IHC [% pos. cells]	unrestricted Ct-value
1	+	100	+	−	2	−	−	1	−
2	+	80	−	+	30	−	−	1	−
3	+	100	+	−	100	+	−	1	+
5	+	100	+	+	100	+	−	30	−
6	+	90	+	+	0	+	−	30	+
7	+	98	−	+	95	+	−	1	−
8	+	100	−	−	100	−	−	40	−
10	+	90	−	+	70	−	−	1	−
11	+	100	+	+	100	+	−	1	+
12	+	90	+	+	80	+	−	1	+
14	+	100	+	+	100	+	−	15	+
15	+	100	−	−	100	−	−	1	−
16	+	60	+	+	80	+	−	30	+
17	+	100	+	+	100	−	−	30	+
19	+	95	+	+	70	+	−	30	+
20	+	100	+	+	100	+	−	40	+
21	+	100	−	+	95	−	−	1	−
22	+	100	+	+	100	+	−	40	+
23	+	90	+	−	1	+	*	40	+
24	+	80	−	+	80	−	−	1	−
25	+	95	+	+	40	+	−	40	+
26	+	100	+	+	100	+	−	1	−
27	+	100	−	+	70	+	−	40	−
28	+	90	+	+	80	+	−	1	+
29	+	70	+	+	40	+	−	40	+
30	+	100	+	+	95	+	−	30	+
31	+	90	−	+	40	−	−	30	−
32	+	100	+	+	85	+	−	10	+
I	+	60	+	−	75	−	−	10	+
II	+	90	+	+	45	−	−	10	−
III	+	90	+	+	90	+	−	10	+
IV	+	90	+	+	60	+	−	30	+

The Oncotype Dx scores shown in Table 2 were allocated according to the respective thresholds (ER: neg. < 6.5, pos. ≥ 6.5; PR: neg. < 5.5; pos. ≥ 5.5; Her2: neg. < 10.7, 10.7 ≤ equivocal < 11.5, pos. ≥ 11.5), whereas the RT^2^-Profiler Ct values were defined as positive if Ct ≤ 35 and as negative if Ct > 35. The table reveals that with one exception the Oncotype Dx defines all analyzed samples as ER positive and Her2 negative regardless of IHC. Regarding the comparison of IHC and RNA detection the Oncotype Dx shows a correlation of 37.5% and the RT^2^-Profiler assay of 71.9%. The inter-assay correlation based on IHC confirmation is about 72% for ER and PGR, and 40.6% for Her2.

### Analysis of the oncotype DX recurrence score formula

The next aim of our study was to understand the influence of the single group scores on the overall RS. The formula (Fig. 2b) was taken from the earlier publication of Paik and colleagues^1^, and it is obvious that due to the factors included in the formula the proliferation group score and the GRB7 group score are the most influential scores.

According to the published rules of Recurrence Score (RS) calculation a proliferation group score below 6.5 is set to 6.5, and a GRB7 group score below 8 is set to 8, although the reason for this procedure is never explained in detail.

**Figure 3:**
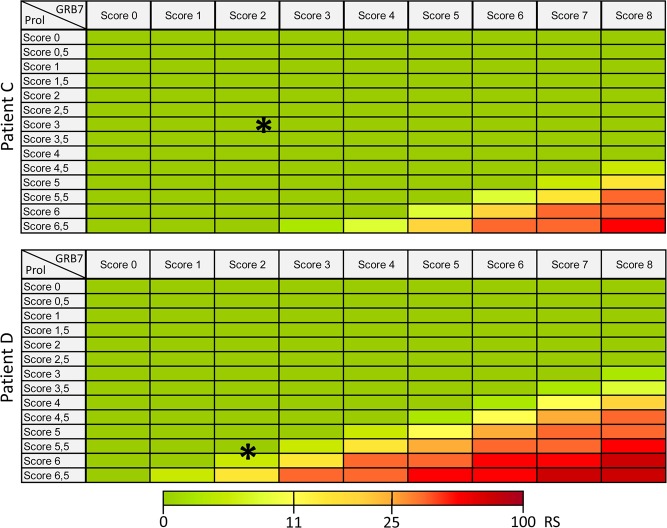
Heatmaps with hypothetical score changes calculated with normalized Ct values (target gene – HKG) and the published formula for the RS (Fig. 2b). The heatmaps of patients C and D show that the adjustment of the GRB7 and the Prol score to 8 and 6.5, respectively, has an overall impact and leads in case of the presented patients to high risk recurrence scores although both patients would belong to the low risk group regarding their original GRB7 and Prol score data (*).

In order to understand the underlying mathematical procedures and to analyze the influence of both group scores on the overall RS, we have taken the normalized Ct values of two randomly selected patients (Patient C and Patient D) and generated a heat-map in which the Ct values other than the GRB7 and the proliferation group score were kept constant as measured and systematically varied the GRB7 and the proliferation group score. In both cases, the patients would be grouped into the high risk group if the respective group scores are adjusted as demanded by the RS calculation procedure (Fig. 3). Because of its impact on the overall RS an explanation why an adjustment of the GRB7 and the proliferation group score is necessary should have been given. However, it was impossible to analyze the entire formula used for the calculation of the recurrence score, because we were unable to identify the publication to which the authors referred in the supplement to the original study [supplement to^1^]. This publication was cited in the supplement as “to be published” and should have shown the exact way how to deduce the formula to calculate the RS.

### Hypothesized score correlations by calculation and comparison of RT^2^ RS with Oncotype Dx RS

**Figure 4:**
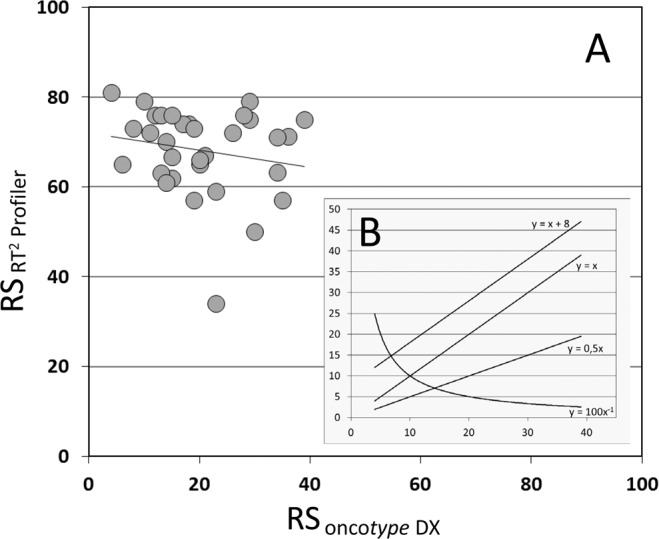
Correlation between Recurrent Scores obtained from the Oncotype Dx and the RT^2^-Profiler. Comparison of RS scores from Oncotype Dx reports versus RT^2^-Profiler RS scores show that there is not any correlation using linear regression analysis (**A**). (**B**) shows hypothesized regression curves that could have been expected.

Based on the normalized Ct_target gene – HKG_ values of the 16 breast cancer related genes included in the Oncotype Dx assay analyzed with the RT^2^ profiler assay, the RT^2^-Profiler RS were calculated by applying the calculation procedure published by Paik and coworkers^1^. Afterwards, the RT^2^-Profiler RS were plotted versus the Oncotype Dx recurrence scores in a Cartesian coordinate system (Fig. 4A). It is not surprising that we did not observe any perfect match between the both recurrence scores, but also an overall correlation could not be observed. It was expected that a correlation, which is ideally presented by the linear function y = x, or its variants by a parallel shift (e.g. y = x + 8) or an altered slope (e.g. y = 0.5 × ), could have been identified (Fig. 4B). As this was not the case, it was hypothesized that at least the two recurrence scores correlate in a nonlinear manner (e.g. y = 100x^−1^). Instead the actual regression grade indicates that no obvious correlation exists between the results of the two assays.

In this context the problem arose that the normalization procedure is not clearly described. On the one hand in the supplement of the underlying publication^1^ it is said that normalization is done by subtracting the average Ct values of the reference genes from the target gene values, whereas the international patent WO2006/052862A1 (p.25) defines normalization as average Ct_reference genes_ − Ct_test gene_. On the other hand the authors refer in the methodological part of the above mentioned publication to the normalization method (2^ΔCt) + 10 published in 2004, where ΔCt equals Ct_test gene_ − Ct_mean of reference genes_^4^, which is also stated in the international patent WO2014/130617A1 p.23. But the same author used the mean cycle threshold of the reference genes minus the mean of triplicate measurements of the target genes as normalizing procedure during analytical validation of the Oncotype Dx in 2007^5^.

In order to further evaluate if any correlation between the RT^2^ Profiler values and the Oncotype Dx results can be attained we applied the different normalization methods described to our expression data (Table 4), but still could not reach the Oncotype RS.

**Table 4:** Comparison of calculated RS scores based on RT^2^-Profiler Ct-values normalized with three different methods published in different contexts with Oncotype Dx assay performance.

Patient	Target Gene-HKG	HKG-Target Gene	(2^ΔCt^) + 10	OncotypeDX
	≤35 cycles	≤35 cycles	≤35 cycles	
1	79	74	5	29
2	79	73	0	10
3	50	100	100	30
5	70	83	52	14
6	73	80	100	8
7	74	79	0	18
8	75	78	0	29
10	77	76	0	12
11	59	94	100	23
12	65	88	100	6
14	67	86	100	15
15	75	77	0	39
16	62	91	0	15
17	71	82	100	36
19	67	85	100	21
20	34	91	100	23
21	74	79	0	17
22	64	89	100	13
23	57	96	100	35
24	76	77	0	13
25	63	90	100	34
26	71	81	100	34
27	73	80	0	19
28	61	92	100	14
29	76	77	100	15
30	65	88	100	20
31	76	76	100	28
32	81	71	39	4
I	72	81	100	26
II	72	79	7	11
III	66	87	100	20
IV	57	95	100	19

### Repeated testing of patient samples

In order to confirm the high intra-assay reproducibility of the Oncotype DX assay, two randomly chosen patient samples (Patient E and Patient F) with known Oncotype DX RS were double pseudonymized given with a different patient history and sent again to Genomic Health for retesting. As the investigations had to be paid by our institutional budgets the number of replicates was limited to these two patients. The reports from Genomic Health in turn were directly delivered to our hospital.

**Figure 5:**
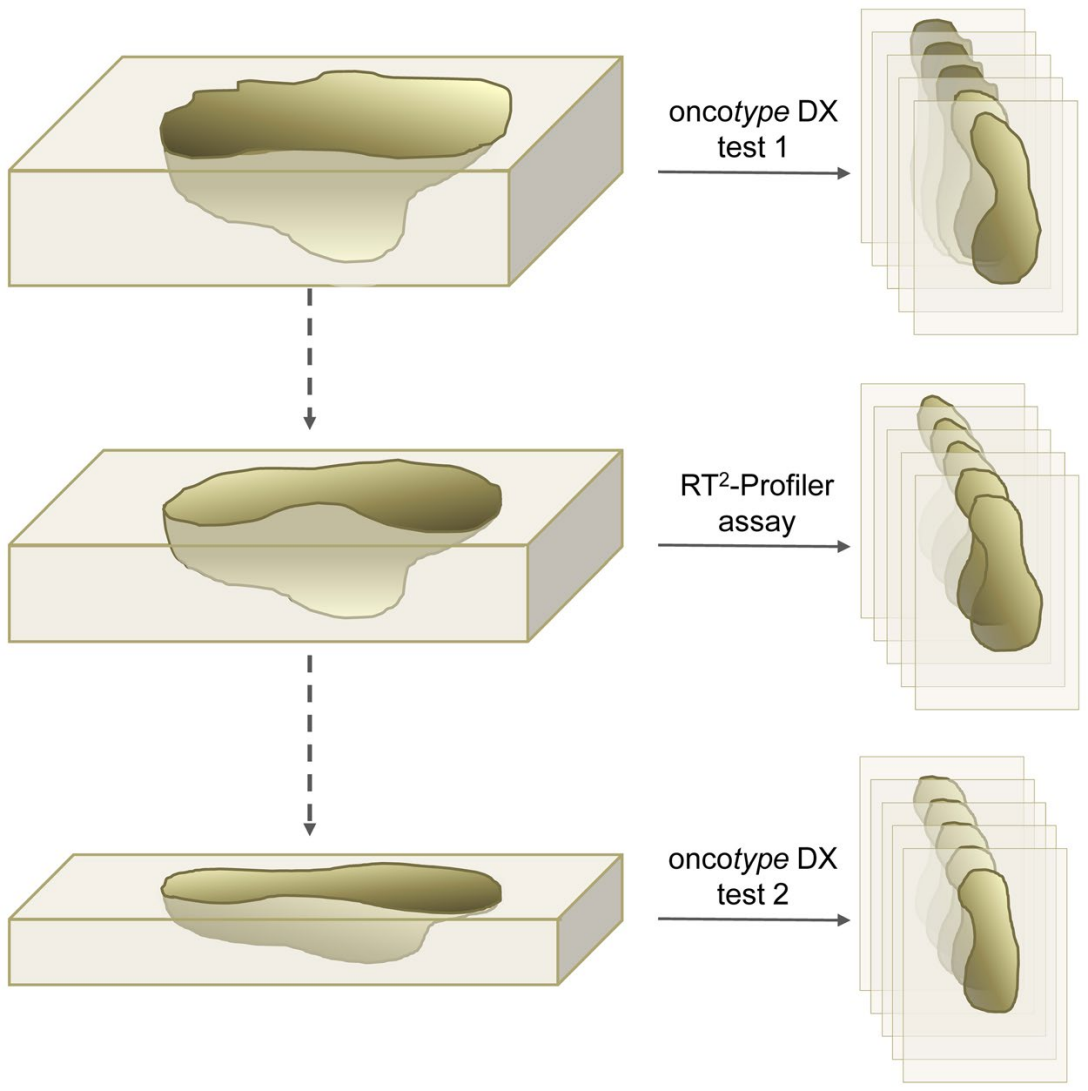
Scheme of a paraffin tissue block undergoing molecular pathology analyses processing. The scheme illustrates that even despite proper processing the analyzed samples are subject to certain changes caused by the morphological (and genetic) intratumor heterogeneity.

At first glance the results confirm the high reproducibility of the Oncotype DX assay as the reported RS values were identical in both investigations for both patients and the reported scores of the control genes differed only slightly, but single gene expression variance cannot be assessed as long as information about the remaining 18 genes is not disclosed. Patient E had an ER score of 11.8, PR score of 9.1, GRB7 score of 10, and a RS score of 4 in the first report, followed by an ER score of 11.8, PR score of 9.2, GRB7 score of 10, and a RS score of 4 in the second report. Patient F had an ER score of 7.9, PR score of 5.5, GRB7 score of 9.4, and a RS score of 28 in the first report, followed by an ER score of 7.3, PR score of 5.5, GRB7 score of 9.5, and a RS score of 28 in the second report. As we prepared RNA of intermediate tumor sections regarding the Oncotype DX testing (Fig. 5) we assumed that at least these RT^2^-Profiler Recurrence Scores should resemble the Oncotype DX results, but based on our data we received RSs of 76 (Patient F) and 81 (Patient E).

Based on the medical report we tried to calculate the Oncotype RSs and consequently failed, because the three listed scores represent only expression levels of the single genes estrogen receptor (ER), progesterone receptor (PR), and HER2. Instead of Ct values that could be inserted into the Oncotype DX formula and that are commonly used to describe the quantity of detected mRNAs the term “score” is used. If these values are just normalized Ct values the term “score” is misleading as it is also used for the group scores in the entire collection of Oncotype assay studies. This applies especially in the case of the ER as it is not clear if the normalized Ct value (score?) used in the RS calculation formula equals the ER score listed in the medical report, which in turn is different from the ER group score. Additionally, it was impossible to retrace the calculation as the Ct values and/or scores based on the mRNAs of the remaining 18 genes are totally missing in the report.

**Figure 6:**
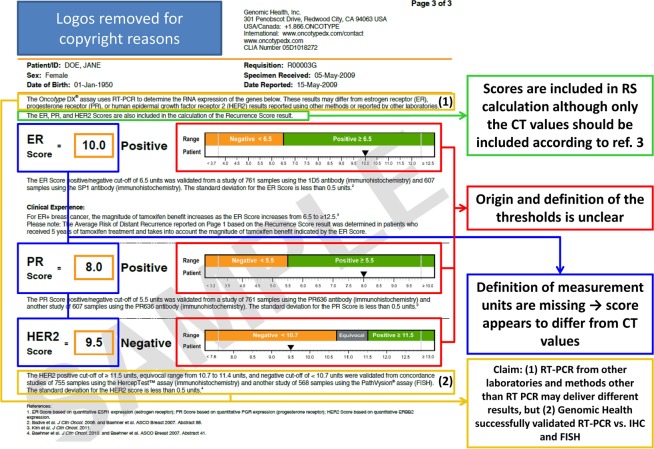
Representative example of an Oncotype Dx report. The sample report downloaded from Genomic Health represents the former version not adapted to the results of the TAILORx study, but the highlighted discrepancies still appear in the novel reports. It is claimed that the single gene expression scores of ER, PR and HER2 are included in the RS calculation (green), although only normalized Ct values were described to be included in the RS calculation. Moreover, single gene expression scores lack an internationally accepted unit (blue) and the origin of the thresholds (red) remains unclear even if all references in the entire citation chain were followed, starting with the references mentioned in the report’s footnotes. Whilst on the report’s top it is mentioned that other methods or assays from other laboratories may differ from the results reported, it is claimed on the bottom that the Her2 score was evaluated against two other methods (yellow).

Because of this finding we took a closer look into Oncotype Dx reports (Fig. 6). Besides the fact that the “quantitative single gene-report” in fact just itemizes three single gene scores, these scores with their respective cut-offs are based on comparison studies with immunohistochemistry or FISH, although in the same report it is stated that the methods used to generate the Oncotype DX reports cannot be compared to other methods.

Whilst we identified the publications in which the respective thresholds were used for the first time, the trace of citations ends in 2004 without any detailed mathematical explanation of the respective threshold determination. Finally, the report contains the information that the PR (progesterone receptor) score is based on the quantitative expression of PGR (progesterone receptor), which allows the conclusion that the listed scores in the report do not equal the normalized Ct values used for RS calculation.

## Discussion

Driven by continued requests for Oncotype DX testing of node negative breast cancer samples and the lack of proper reimbursement until the third quarter of 2019 we aimed to setup an in-house assay that could be used to reproduce the Oncotype DX breast cancer scoring methodology in our hospital.

Despite the statement of Genomic Health that Oncotype results may differ depending on laboratory and method, we hypothesized that qPCR assays like a customized RT^2^-Profiler assay, which is based on RT-PCR methods, should be able to deliver results similar to the Oncotype assay according to the criteria of Nosek and Errington^2,3^, as the Oncotype DX is also based on RT-PCR techniques. We also hypothesized that it should be possible to estimate the recurrence risk by calculating the RS with data obtained by the RT^2^-Profiler assay multiplied with or by adding a correction factor obtained from a regression grade resulting from the application of Oncotype Dx RS versus RT^2^-Profiler scores in a Cartesian coordinate system.

For this reason the original study in which the Oncotype Dx assay was published provided the basis for our pilot study. In order not to miss any important assay information we double checked the published data with the international patents specifications (International Patent Publication Numbers WO2006/052862A1 and WO2014/130617A1), which confirmed the formula to calculate the RS. With regard to our expectation that a high Oncotype Dx RS score would result in a high RT^2^-Profiler RS score, whilst a low score of the Oncotype Dx assay should have led to a low RT^2^-Profiler score, we discovered that neither the recurrent scores nor the reported ER, PR and Her2 scores were congruent between both methods nor displayed the same general outcome. In contrast, no correlation was observed at all, which leads to the uncomfortable conclusion that no inter-assay concordance between the Oncotype DX and the RT^2^-Profiler method exists. Because we are aware of the fact that discrepancies may be attributable to differences during overall assay performance and data acquisition, we analyzed all steps in the Oncotype testing that we were able to follow to evaluate the impact of the single parameters. This in turn revealed some intra-assay related ambiguities, which besides the lack of reported data impede reproducibility.

In the *Cancer Biology Reproducibility Study* the Errington group justifiably demands that retesting a hypothesis with the same or a closely related methodology should provide the same evidence that in turn enables the investigator to converge on an explanation for the finding that is not dependent on either methodology^3^. This inter-assay reproducibility was obviously not fulfilled for the Oncotype DX in comparison to the RT^2^-Profiler assay, which is due to the fact that the Oncotype DX assay remains a black box. Several parameters are not reported at all, while other parameters are reported in a way that differs from all claims in the original publication and the patent files available for the assay (International Patent Publication Numbers WO2006/052862A1 and WO2014/130617A1), as shown in the results.

Paik and coworkers, however, have reported that their assay is able to detect 21 mRNAs from FFPE tumor tissues whose normalized Ct values can be used to calculate 4 group scores that in concert with some non-grouped genes form the basis for the recurrence score^1^. Thereby, the formula is based on training set data from 3 clinical trials. When reading this earlier publication it became obvious that these trials were not published as peer reviewed full articles in advance of the underlying study from 2004 but as posters for scientific conferences, that therefore are no longer available (Refs.^24^–^26^ in Paik *et al*., 2004). In addition, it was declared that the formula was based on gene-expression datasets including 250 genes that were subjected to correlation analysis, dimension reduction, Martingale residual analysis, concordance measures of accuracy, and bootstrap resampling. Although the details of this methodology were claimed to be published in detail separately (supplement to^1^), we have not yet identified the publication that included these details, thus also this part of the methodology remains unclear and is also not mentioned in the corresponding patent file, not even as confidential. This is a core issue, as besides the striking points mentioned above, the question arises what has to be done if the RS becomes negative. Indeed it is defined that RS = 0 if RSu < 0, but simultaneously RS = 20 × (RSu-6.7) if 0 ≤ RSu ≤ 100 is applied ((1), p. 2819). Even though we are no mathematicians, we think that the terms should rather be RS = 0 if RSu ≤ 6.7 and RS = 20 × (RSu-6.7) if 6.7 ≤ RSu ≤ 100, as otherwise negative recurrence scores could occur that would not be covered by the 0–100 Recurrence Score scale. On the other hand it is stated that RS = 100 if RSu > 100 but the RS already becomes 100 or greater if RSu ≥ 11.7. These published statements were corrected in WO2006/052862A1 and replaced by a mathematically correct basic assumption. For this reason it is all the more surprising that the above mentioned formulas are listed again for RS rescaling in WO2014/130617A1 (p. 32). In this context it would be interesting to compare patient data of same RS. As the RS results from weighted gene expressions values of genes with different function, it is possible that a significant upregulation of a distinct gene group may be compensated by regulatory effects of other genes leading to the same recurrence score achieved by a moderate upregulation of a third functional group in another patient sample. For this reason the raw data generated by Genomic Health could be very useful to understand the biology of breast cancer in more detail.

In addition, rather than exact matches we expected that repeated testing would result in RS scores similar to each other within the range of the reported standard deviation^1^, as the majority of tumors are not homogenous masses, but are subject to intra-tumor differences, even in the so called “homogeneous” tumors. Gyanchandami and coworkers in this context concluded that the interpatient tumor heterogeneity is higher than the intra-tumor heterogeneity, but that also the intra-tumor heterogeneity may lead to an over- or underestimation of gene expression profiles^6^. As the first Oncotype DX testing as well as the subsequent RT^2^-Profiler testing and the second Oncotype testing were performed on the same FFPE tissue blocks, but on different tissue sections, there should have been at least moderate intra-tumor heterogeneity within the standard deviation range. Regarding our evaluation samples, which were analyzed in quadruplicate we received RT^2^-Profiler mean recurrence scores of 72 with SD = 1.7 for one patient and 72 with SD = 4.7 for the other, whereas the identical results of the two patients retested with the Oncotype DX are absolutely impressive. Although the Oncotype DX assay appears to be highly reproducible within its black box, neither clinicians nor patients get access to any raw data.

The access to raw data also could be useful to explain the observation that a remarkable number of genes display high Ct values or are not expressed if measured with the RT^2^-Profiler assay. It was hypothesized that these high Ct values may be a result of the low RNA input of 100 ng, but as there is no information available how much RNA is used in the Oncotype Dx we worked with the indicated RNA limit of 500 ng leading in several cases to inappropriate Ct values between 35 and 40, which could also have influenced the subsequent recurrence score calculations. Nevertheless, without any published raw data a normalized gene value of 0 in the Oncotype Dx 0–15 scaling system may result from subtracting Ct 34 _(target gene)_ − Ct 34 _(HKG)_ as well as from Ct 12 _(target gene)_ − Ct 12 _(HKG)_.

The novel version of the Oncotype DX medical report includes a short statement that the reported single gene expression scores are for quality control purposes, but it is not defined if they only confirm the inclusion criteria of a Her2 negative ER positive status of the investigated tumor or if the controls provide information about assay performance. Additionally, these scores are also included in the calculation of the RS, although normalized Ct values should be used the formula.

Thereby the question arises if the term “score” is used as a synonym for “normalized Ct value” or if it describes a different weighted value (Fig. 6). Besides the fact that the wording ‘score’ could be misleading and draw the report recipients’ attention to the groups score that were extensively discussed by Paik *et al*.^1^, any commonly accepted unit describing the gene expression on the mRNA level is missing. If the ER score, the PR score, and the Her2 score are equal to the RT-qPCR result, the applicable unit would be a (normalized) Ct value or a copy number that refers to a reference value such as volume (e.g. ml tissue lysate) or weight (e.g. gram of tissue, which would be in accordance to international standards^7^. In this context it is worth noting that the Merriam Webster dictionary defines “score” (https://www.merriam-webster.com/dictionary/score) as a “number that expresses accomplishment (as in a game or test) or excellence (as in quality) either absolutely in points gained or by comparison to a standard” or “a group of…things”. In contrast, “value” (https://www.merriam-webster.com/dictionary/value) is defined as “a numerical quantity that is assigned or is determined by calculation or measurement”. Moreover, the footnotes of the report states that on the one hand the expression profiles of ER, PR and Her2 may differ from results obtained with other methods in other laboratories, but on the other hand it is claimed that the RT-PCR was validated against exactly those other methods with a high concordance^8–15^. To our surprise, whilst the literature cited in the footnotes mentions that the respective cut-off values are derived from comparison studies with other methods, none of the cited studies explains what the terms ER score, the PR score, and the Her2 score exactly mean, and the cut-off values were just claimed to be predefined. When following the citation chain, starting with the references mentioned in the Oncotype Dx reports^8–15^, one ends up at the study of Esteva and coworkers^8^, which to our knowledge was the earliest study that used the respective cut-off values. In this study it is only claimed that the cut-off points were used “as established on the basis of analysis of clinical results from prior studies”, but it remains uncited which studies these are. Although we are experienced in RT^2^-Profiler analysis^16^ and transcriptome analysis^17^ and although the internal controls delivered proper results, we have to accept that the RT^2^-Profiler assay cannot be used to confirm the Oncotype DX, but so far several studies have shown that even the core conclusions of the Prosigna, the MammaPrint, the Oncoptype Dx, and the EndoPredict assays have only moderate agreements^18–23^, which is anything but acceptable as therapy decisions depend on the obtained results.

That at least an independent intra-assay reproducibility as prerequisite for *in vitro* diagnostics is possible was shown for the Prosigna assay by Nielsen *et al*.^24^ as well as for the EndoPredict^25^ and the MammaPrint assay by Marchionni and colleagues^26^. In this context, Marchionni and coworkers reported that, due to the unavailability of the original data, it was not possible to carry out this process for OncotypeDX, which is presently the most used and validated predictor of this kind^26^. This finding is confirmed by our experiences in the present study.

Although our study is not, however, intended to discuss any clinical application of the Oncotype DX, we are aware of the fact that it will be argued that the conclusions from the TAILORx study confirm the clinical utility of the Oncotype Dx assay^27^.

From this latter study it was concluded that adjuvant endocrine therapy and chemoendocrine therapy had similar efficacy in women with hormone-receptor–positive, HER2-negative, axillary node–negative breast cancer who had a midrange 21-gene recurrence score^27^, whereas some benefit of chemotherapy found in women 50 years of age or younger with a recurrence score of 16 to 25.

But in order to draw the conclusion that specifically the Oncotype DX assay is essential for the stratification of patients, a control group without Oncotype DX RS result, but stratified by clinical, histopathological, and immunohistological diagnostics, should have been included within the scope of a matched pair study.

However, due to the fact that an Oncotype Dx result was the inclusion criterion for the TAILORx study accompanied by the fact that a control group is missing the TAILORx study shows that risk stratification is beneficial, but it remains open if this stratification benefits exclusively from the Oncotype Dx. For the present study no conclusions on the clinical utility and necessity of either the Oncotype Dx recurrence score or the RT^2^-Profiler Recurrence score can be drawn, as the included patients were diagnosed in 2016 and 2017. Thus the five year prediction interval for which the Oncotype Dx Recurrence score is used is still ongoing. Consequently this issue needs to be addressed in a separate study.

In contrast, some studies have impressively shown that the combination of good clinical observations, phycian’s experience, and sophisticated pathological methods of histomorphological screening and immunohistochemistry are sufficient to stratify patients and enable therapy decisions even without cost intensive molecular assays^28–31^.

However, due to the complexity of the published datasets, the fact that key informations appear to be unpublished in peer reviewed papers, and due to the lack of transparency of the Oncotype DX results it is neither possible to confirm the Oncotype DX nor to optimize our RT^2^-Profiler assay. Although all past Oncotype Dx studies including the TAILORx study show that the assay delivers appropriate likelihoods of recurrence, it remains to be discussed if this is acceptable for an assay that affects therapy decisions in the daily routine. Maybe things will straighten out, if samples can be retested decentralized with an Oncotype Dx adapted Biocartis Idylla System that is to be launched (https://www.biocartis.com/about-us/our-partners; https://newsroom.genomichealth.com/news-releases/news-release-details/genomic-health-and-biocartis-expand-collaboration-urology).

## Supplementary information

Supplementary Information

Supplementary Information 2

Supplementary  Information 3
