# Supplementary material for: Oncotype DX Breast Cancer recurrence score resists inter-assay reproducibility with RT2-Profiler Multiplex RT-PCR
Source: Sci Rep. 2019 Dec 30;9:20266. doi: 10.1038/s41598-019-56910-0 (PMC6937305; doi:10.1038/s41598-019-56910-0)
Supplement: Supplementary file 1 — Supplementary Information [file 41598_2019_56910_MOESM1_ESM.doc]

**Oncotype DX Breast Cancer recurrence score resists inter-assay reproducibility with RT^2^-Profiler Multiplex RT-PCR**

Verena Schildgen^1^, Mathias Warm^2^, Michael Brockmann^1^, and Oliver Schildgen^1^

**Authors affiliations:**

1. Kliniken der Stadt Köln gGmbH, Klinikum der Privaten Universität Witten/Herdecke, Institut für Pathologie, Ostmerheimer Str. 200, D-51109 Köln, Germany
2. Kliniken der Stadt Köln gGmbH, Brustzentrum, Neufelder Str. 34, D-51067 Köln, Germany

**Send correspondence to:**

Prof. Dr. rer. nat. Oliver Schildgen

Dipl.-Biologe, Fachvirologe GfV

Institut für Pathologie

Kliniken der Stadt Köln gGmbH

Klinikum der Privaten Universität Witten/Herdecke mit Sitz in Köln

Ostmerheimer Str. 200

D-51109 Köln (Cologne)

Germany

Tel.: +49(0)221-890713467

Fax: +49(0)221-89073542

E-Mail: [schildgeno@kliniken-koeln.de](mailto:schildgeno@kliniken-koeln.de)

E-Mail: [oliver.schildgen@uni-wh.de](mailto:oliver.schildgen@uni-wh.de)

**Legends for supplements**

**Supplement 1:** Excel file containing RT^2^-Profiler assay design’s detailed gene list and order numbers as provided by Qiagen.

**Supplement 2:** Excel file containing Ct values and calculations of all patients included in the study. Normalization was done by subtracting the HKG from the target gene. Ct values >35 are defined as negative and were not considered for calculation.
